# Consumer Decision-Making and Ophthalmic Conditions: A Novel Area of Marketing Research

**DOI:** 10.22336/rjo.2026.26

**Published:** 2026

**Authors:** Consuela-Mădălina Gheorghe

Understanding how psychological, social, cultural, and economic factors affect customer decision-making has historically been a focus of marketing. However, visual health is a crucial aspect that has received very little attention. Ophthalmic disorders impact billions of people of all ages and are one of the major causes of impairment globally. Since vision is the primary sensory modality involved in information acquisition during purchasing decisions, impairments of visual function have profound implications for consumer behavior, brand communication, digital marketing, and public policy. More scientific research and interdisciplinary cooperation are needed to address this new nexus between marketing and ophthalmology.

Visual stimuli are becoming increasingly important in today’s economy. Visual perception plays a major role in digital ads, product packaging, smartphone applications, websites, augmented reality, and social media platforms. The significance of visual cues in drawing attention, facilitating information processing, and influencing purchasing behavior is emphasized by marketing theories such as the Information Processing Model (Bettman JR. An Information Processing Theory of Consumer Choice. 1979, Boston, Addison-Wesley), Dual Coding Theory (Paivio A. Mental Representations: A Dual Coding Approach. 1990, Oxford University Press. doi: https://doi.org/10.1093/acprof:oso/9780195066661.001.0001), and the Elaboration Likelihood Model (Petty RE, Cacioppo JT. Communication and Persuasion: Central and Peripheral Routes to Attitude Change. 1986, New York, Springer-Verlag. doi: http://dx.doi.org/10.1007/978-1-4612-4964-1). Yet these theoretical frameworks often assume normal visual performance, overlooking the considerable minority of consumers living with ophthalmic conditions.

The World Health Organization (World Health Organization. World Report on Vision. 2019) estimates that at least 2.2 billion individuals worldwide have blindness or vision impairment, and at least 1 billion of these cases could have been prevented or remain untreated. The prevalence of age-related eye illnesses—including cataracts, glaucoma, diabetic retinopathy, and age-related macular degeneration (AMD)—continues to rise due to population aging and increasing rates of chronic diseases such as diabetes (Flaxman SR, Bourne RRA, Resnikoff S, et al.; Vision Loss Expert Group of the Global Burden of Disease Study. Global causes of blindness and distance vision impairment 1990-2020: a systematic review and meta-analysis. Lancet Glob Health. 2017 Dec;5(12):e1221-e1234. doi: 10.1016/S2214-109X(17)30393-5. Epub 2017 Oct 11. PMID: 29032195; Burton MJ, Ramke J, Marques AP, et al. The Lancet Global Health Commission on Global Eye Health: vision beyond 2020. Lancet Glob Health. 2021 Apr;9(4):e489-e551. doi: 10.1016/S2214-109X(20)30488-5. Epub 2021 Feb 16. PMID: 33607016; PMCID: PMC7966694). As a result, an increasing number of consumers find it challenging to read labels, identify brands, compare prices, decipher internet interfaces, and assess visual advertising.

One aspect of functional vision pertinent to marketing is visual acuity. Contrast sensitivity, color discrimination, peripheral vision, depth perception, and ocular movement all contribute to the efficient processing of marketing information. For instance, cataracts impair color perception and contrast sensitivity, making goods packaging appear faded or hazy. Peripheral vision is gradually limited by glaucoma, which may make it more difficult for customers to navigate shop spaces or see items outside their field of vision. Because AMD impairs central vision, it becomes more challenging to read nutritional labels, product information, and digital marketing (Lim LS, Mitchell P, Seddon JM, Holz FG, Wong TY. Age-related macular degeneration. Lancet. 2012 May 5;379(9827):1728-38. doi: 10.1016/S0140-6736(12)60282-7. PMID: 22559899). Decision fatigue, decreased confidence in purchase judgments, and altered purchasing efficiency are all possible outcomes of these impairments.

There are more difficulties with digital marketing. Electronic commerce depends largely on visual interfaces that often include small fonts, low-contrast color schemes, moving visuals, and sophisticated navigation frameworks. When interacting with online retailers, consumers with low vision may have to exert more cognitive effort, which could lead to lower purchase intentions, more cart abandonment, and decreased trust in digital platforms. Research on human-computer interaction has repeatedly shown that accessibility has a major impact on user happiness and behavioral intentions (Norman D. The Design of Everyday Things. Revised and Expanded Edition, 2013, New York, Basic Books; Lazar J, Goldstein DF, Taylor A. Ensuring Digital Accessibility through Process and Policy. 1st Edition, 2015, Morgan Kaufmann). As a result, visual accessibility should now be considered a strategic element of customer experience management rather than just a legal need.

Neural mechanisms underlying consumer decision-making may be impacted by visual impairments, according to emerging findings from neuromarketing. Eye-tracking technologies, frequently applied in marketing research, have revealed that gaze duration, fixation patterns, and visual attention greatly influence purchasing behavior (Wedel M, Pieters R. Visual Marketing: From Attention to Action. First Edition, 2007, New York, Psychology Press. doi: https://doi.org/10.4324/9780203809617). However, ocular illnesses fundamentally change these visual scanning patterns. Patients with glaucoma, AMD, or diabetic retinopathy exhibit altered eye movements, longer fixation durations, and compensatory scanning strategies, potentially affecting their exposure to marketing cues and subsequent purchase decisions. Integrating ophthalmological assessments into neuromarketing studies could therefore increase our understanding of varied customer responses.

Particular consideration should be given to color perception. One of the most powerful factors influencing brand identification and emotional communication is color. However, hundreds of millions of people worldwide suffer from hereditary and acquired color vision deficits. Customers who are color vision impaired may misunderstand brand differentiation tactics, safety warnings, promotional signals, or product classifications. Despite established guidelines for accessible color combinations and universal design principles, marketing professionals seldom take these constraints into account when designing packages or creating advertisements (World Wide Web Consortium (W3C). Web Content Accessibility Guidelines (WCAG) 2.2. 2024).

The aging population further reinforces the significance of ophthalmic issues in marketing. The fastest-growing consumer sector in the world is older folks, who also have the largest prevalence of visual impairment. Marketing initiatives targeting older consumers usually highlight healthy aging, financial planning, medications, or medical services, but often omit age-related losses in visual function. Larger typography, increased contrast, streamlined layouts, intuitive navigation, and multimodal communication could considerably improve both marketing effectiveness and customer autonomy.

Artificial intelligence promises to tailor marketing communications based on visual skills. Adaptive interfaces that automatically adjust font size, contrast, brightness, image magnification, and voice-assisted navigation may remove barriers for visually challenged users. Advances in computer vision and machine learning could enable personalized digital experiences that simultaneously enhance accessibility and customer satisfaction. Such advances coincide with the broader drive toward inclusive marketing and universal design.

From an ethical perspective, marketers have a responsibility to guarantee that visual impairments do not become barriers to informed customer choice. Research on health literacy has consistently shown that poor decision-making and health outcomes are caused by inadequate access to comprehensible health information (Nutbeam D. Health literacy as a public health goal: 25 years on. Health Promot Int. 2025 Jul 1;40(4):daaf119. doi: 10.1093/heapro/daaf119. PMID: 40665800; Sørensen K, Van den Broucke S, Fullam J, Doyle G, Pelikan J, Slonska Z, Brand H; (HLS-EU) Consortium Health Literacy Project European. Health literacy and public health: a systematic review and integration of definitions and models. BMC Public Health. 2012 Jan 25;12:80. doi: 10.1186/1471-2458-12-80. PMID: 22276600; PMCID: PMC3292515). In the context of pharmaceutical marketing, medical device communication, and healthcare service promotion, visual accessibility is a crucial—yet often disregarded—aspect of health literacy. When customers are making decisions about pharmaceuticals, dietary supplements, medical devices, or ophthalmic treatments, clear visual communication is especially crucial.

Future studies should use multidisciplinary methods that incorporate digital marketing, consumer psychology, behavioral economics, ophthalmology, and neuroscience. Several research priorities emerge: (1) quantifying the effects of specific ophthalmic conditions on purchasing behavior; (2) evaluating visual accessibility across online retail platforms; (3) investigating eye disease-related differences in neuromarketing metrics; (4) developing predictive models of consumer behavior incorporating visual function; and (5) establishing evidence-based guidelines for visually inclusive marketing communication.

In conclusion, eye disorders should be regarded not only as medical issues but also as factors that influence consumer involvement in more visual marketplaces. The number of consumers impacted by visual impairments will continue to rise as societies age and digital commerce grows. Therefore, the conceptual framework of marketing science needs to be expanded to incorporate visual health as a key factor influencing customer behavior. Such integration will not only increase academic knowledge but also encourage more equal, accessible, and socially responsible marketing methods that benefit both firms and consumers.

**Senior Lecturer, PhD, Philologist, Certified translator**

